# LAWS, REGULATIONS, AND POLICY: New Food Safety Law Brings Opportunities Amid Hurdles

**DOI:** 10.1289/ehp.119-a119

**Published:** 2011-03

**Authors:** David A. Taylor

**Affiliations:** **David A. Taylor** writes for *The Washington Post *and *Smithsonian *and is author of *Ginseng, the Divine Root*, about the science and subculture surrounding the medicinal plant. He teaches science writing at The Writer’s Center in Maryland

When President Obama signed the FDA [U.S. Food and Drug Administration] Food Safety Modernization Act^1^ into law on 4 January 2011, it marked the farthest-reaching changes to the U.S. food safety system in more than 70 years. Crafted in light of concern over foodborne disease outbreaks and perceived underinvestment in food-inspection capacity,^2^ the law combines a mandate for more FDA inspections with authority to respond quickly to illness outbreaks.

The bill fundamentally restructures the way FDA monitors the food supply,” says Caroline Smith DeWaal, director of food safety at the nonprofit Center for Science in the Public Interest. “FDA’s oversight role is greatly enhanced.” The agency also has enhanced authority over imported processors, a growing source of the U.S. food supply. She notes the law sets new standards for on-farm production, particularly certain high-risk fruits and vegetables.^3^ The FDA shares responsibility for food safety with U.S. Department of Agriculture (USDA), which handles poultry, meat, and certain egg products.

On the other hand, the law doesn’t mark a huge change in direction overall. Robert Buchanan, director of the Center for Food Safety and Security Systems at the University of Maryland, explains that the new law gives FDA “some additional powers for when the industry isn’t doing what it’s supposed to be doing.” These include setting limits on key food pathogens, better surveillance, and more capacity for inspections and investigations following illness reports.

The bill lays out a combination of producer performance standards and government inspections designed to ensure those standards are upheld. It sets a goal of “not fewer than 4,000 field staff members in fiscal year 2011 to carry out food-related activities,” according to FDA spokesman Douglas Karas. Last year’s budget allowed for about 2,800 full-time staff for field activities related to food, supplemented by other staff.

Despite the law’s call for more inspectors and more frequent inspections, with the new Republican Congress vowing to slash federal budgets, Buchanan says, “Frankly, USDA and FDA will be lucky to keep the number of inspectors they already have. It’s a real concern.” The FDA declined interview requests, saying it’s still studying the new law and exploring partnerships with other agencies and watchdog groups.

The new law leaves it to industry to decide which technologies to use to track outbreaks and manage tracking data. “Frequent shopper” cards used by grocery stores to track customers’ preferences may help. Some stores’ cards track food lot numbers, and they have led to recalls in some cases, according to David Goldman, assistant administrator for the Office of Public Health Science at the USDA Food Safety and Inspection Service.

Some experts expect what the *Washington Post* called “a small gold rush” among tech companies anticipating a growing market in new food-tracking technologies.^4^ The California-based company YottaMark, Inc., for example, markets the HarvestMark system, which lets consumers scan produce labels using an iPhone app or type in tracking numbers online to learn precisely where the produce was grown and when it was harvested. HarvestMark is currently used by 200 companies.

Will the new law reform the system? Time will tell. “This is always going to be an evolving activity,” Buchanan says. “But do I think this law is going to have a big impact? Yes. Huge.”
